# Occurrence and co-existence of localized musculoskeletal symptoms and findings in work-attending orchestra musicians - an exploratory cross-sectional study

**DOI:** 10.1186/1756-0500-5-541

**Published:** 2012-10-01

**Authors:** Helene M Paarup, Jesper Baelum, Claus Manniche, Jonas W Holm, Niels Wedderkopp

**Affiliations:** 1 Research Unit of Occupational and Environmental Health, Institute of Clinical Research, Faculty of Health Sciences, University of Southern Denmark, Odense, Denmark; 2 Department of Occupational and Environmental Medicine, Odense University Hospital, Odense, Denmark; 3 Department of Occupational Medicine, Koege Hospital, Koege, Denmark; 4 The Research Department, Spine Center of Southern Denmark, Hospital Lillebaelt, Middelfart, Denmark; 5 Institute of Regional Health Services, Faculty of Health Sciences, University of Southern Denmark, Odense, Denmark

**Keywords:** Musicians, Symphony orchestra, Musculoskeletal symptoms, Musculoskeletal findings, Clinical examination, Pain, Repetitive strain injury, Nordic musculoskeletal questionnaire, Cross-sectional, Exploratory

## Abstract

**Background:**

Due to ergonomic exposure musicians are at risk of work-related musculoskeletal disorders in the neck, back, and upper extremities. The literature confirms musculoskeletal problems in these anatomic regions among orchestra musicians.

**Methods:**

An explorative cross-sectional study among 441 musicians from six Danish symphony orchestras; 216 underwent a clinical examination constructed for the purpose. Prior to the examination the musicians rated their maximally perceived trouble within the last week on a scheme blinded to the examiner. Accessibility to the clinical examination differed between orchestras.

The aims were to assess the prevalence of 1) perceived symptoms within the previous week in the neck, back and limbs and of 2) clinical findings in the neck, back, and upper extremities, and 3) to investigate the co-existence of the perceived symptoms and clinical findings.

**Results:**

Symptoms and findings were most common in the neck, back, and shoulders. Due to a poor co-existence between self-reported symptoms and clinical findings musicians experiencing bodily trouble could not be identified through this clinical examination. Free accessibility to the examination was of major importance to participation.

**Conclusions:**

In compliance with the purpose, perceived symptoms within the previous week and present clinical findings were assessed. Although both symptoms and findings were most frequent in the neck, back, and shoulders the co-existence of anatomically localized symptoms and findings was generally quite poor in this study.

Discrepancy between symptoms and findings might be caused by the participants currently attending work and therefore being relatively healthy, and the fluctuating nature of musculoskeletal problems. Furthermore from a comparison of different measuring units - self-reported symptoms being period prevalence rates and clinical findings point prevalence rates; a bias which may also be inherent in similar studies combining self-reported questionnaire data and clinical findings.

## Background

### Musicians at risk of work-related musculoskeletal disorders

Due to playing traditions, the construction and sound of the classical instruments, professional instrumentalists - such as symphony orchestra musicians - are exposed to monotonous, asymmetric, or even awkward working postures. The postures are characterized as giving little scope for variation in the neck and trunk, and they comprise repetitive use of the upper extremity, especially with repetitive precision movements in the fingers [1-5]. Due to this occupational exposure professional musicians can be considered at risk of work-related musculoskeletal disorders in the neck, back, and upper extremities [6-8]. And studies comprising the playing-related biomechanical exposure specifically among professional orchestra musicians have demonstrated an association between the playing-posture and the perceived musculoskeletal symptoms [1,9].

### Musicians’ musculoskeletal disorders

Musculoskeletal problems in musicians, however not only for symphony orchestra musicians, have generally tended to involve the neck, back, or upper extremities [10,11]. Studies of diverse groups of pre-professional and professional musicians have shown very wide ranges of prevalence rates of playing-related musculoskeletal disorders ranging from 26-93% [11,12]. Recent studies of elite musicians playing in professional orchestras have emphasized the extent of the musculoskeletal problems in this occupational group by demonstrating that the majority of the orchestra musicians reported perceived musculoskeletal symptoms. These studies revealed that also in the elite musicians the most affected anatomic regions were the neck, back, and upper extremities, and that females tended to report more symptoms than males [9,13,14]. Studies of professional orchestra musicians have also demonstrated a difference between the instrument groups and have indicated that string players had higher odds ratios for more of the symptoms than (most of) the wood wind players had [1,13,14].

Clinical findings have in particular comprised musculotendinous disorders and entrapment or paraesthesia of peripheral nerves [15,16]. The disorders most often tend to be within the umbrella term repetitive strain injuries (RSI), also known as occupational overuse syndrome or cumulative trauma disorders, which have a wide range of severity from beginning of soreness to persisting pain and functional impairment [17,18]. These disorders are thereby not only widespread but with increasing severity also potentially career-inhibiting.

### Diagnoses – an expression of morbidity

Musculoskeletal disorders have been investigated in different occupational groups by combining perceived symptoms as self-reported data and diagnoses based on findings in a clinical examination [19-23]. Such studies have often demonstrated a gap between perceived symptoms and clinical diagnoses. Among the reflections on why this gap is found is whether the perception of symptoms is the same in work-attending or sickness-absent employees, and whether it is appropriate to add actual diagnoses on employees who are fit enough to attend work. It can be stated that in themselves musculoskeletal diagnoses, although indicating a pathology, do not reveal much about the extent of a problem, as two individuals with the same diagnosis can perceive the severity as well as the impact of it very differently. This difference in perceived symptoms may lead to different health behaviours based on the same clinical diagnosis; among professionals this could e.g. be continued full-time job attendance, medical examination, medical treatment, or reporting in sick. From this approach an examination of work-attending employees maybe should be extended to including simple clinical findings instead of actual diagnoses, in particular because some diagnoses are a categorised expression of morbidity which is not suitable for a fully job-attending study population; furthermore because there can be symptoms and findings that may not be included in a diagnosis.

### A qualitative description of the orchestra musicians’ musculoskeletal problems

Before deciding how to perform the clinical examination, semi-structured focus interviews were conducted with individual symphony orchestra musicians (a string player, a wind player, and a percussionist) as well as a focus group interview with two employee representatives from each participating orchestra and from different instrument groups. The interviews were about the musicians’ health and work and were held to ensure that the survey not only was of interest from the researchers’ point of view but also of relevance to the musicians. The key points of the information obtained through the interviews were: 1) All interviews indicated that high frequencies of musculoskeletal problems were considered common, and most likely most of the musicians were affected. 2) The musculoskeletal problems were described as widespread, most commonly as soreness or pain in the neck, back, and shoulders, and long duration not being unusual. 3) A common opinion was that due to a competitive working environment there had generally been a tendency for the musicians to keep health complaints to themselves, and that this still is a typical approach among some musicians, in some instrument groups more than others and in some orchestras more than others. 4) A common opinion was that playing despite discomfort is normal.

### Deciding the study design

The problems addressed in the interviews – musculoskeletal problems being of high frequency, widespread, and affecting neck, back, and upper extremities – were to a large extent consistent with information found in the literature [9-16]. Therefore the examination was planned to comprise these anatomic regions. As playing despite symptoms was common, perceived pain or soreness before (within the last week) as well as during the examination were included. And - as argued above - it was decided that the examination should comprise symptoms – but not diagnoses. It was also decided that the study should be conducted as a cross-sectional study as - in case of a high frequency of widespread problems – it might be difficult or impossible to find matched controls enough for a nested case–control study. Also it was considered that a tendency of keeping health complaints as private for competitive reasons comprised the risk, that selected cases would feel stigmatized and refuse participation.

### Aims of study

**Figure 1:**
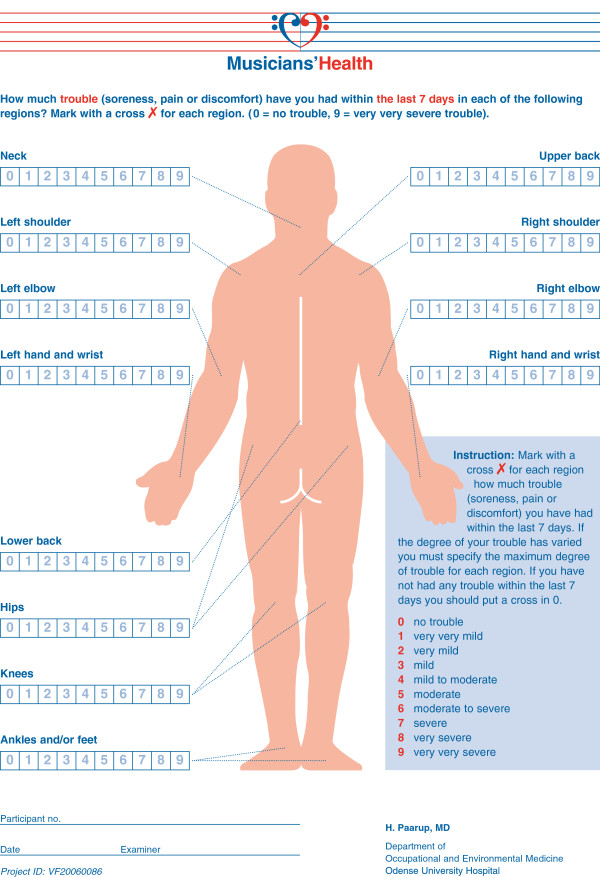
**Rating scheme for maximally perceived trouble within the previous seven days in 12 anatomic regions.**

**Figure 2:**
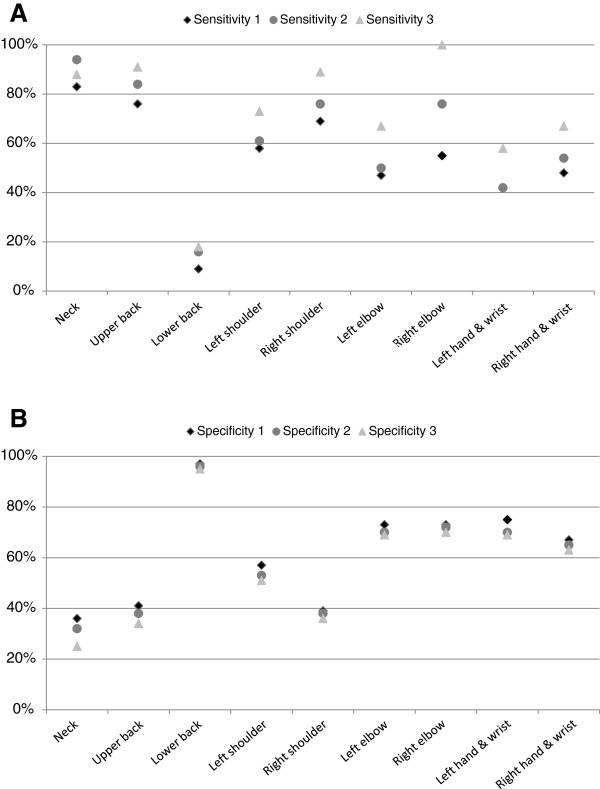
**Sensitivity and specificity of the clinical examination for identifying musicians with perceived musculoskeletal trouble. A**: The overall sensitivity of the test for each of clinically examined region. Sensitivity 1: the sensitivity of the test for participants who reported “any” degree of trouble. Sensitivity 2: the sensitivity of the test for those who reported “moderate or severe” trouble. Sensitivity 3: the sensitivity for musicians who reported “severe” trouble. **B**: The overall specificity of the test for each of clinically examined region. Specificity 1: calculated using “none” or “any” degree of trouble. Specificity 2: calculated using “none/mild” or “moderate/severe” trouble. Specificity 3: calculated using “less than severe” or “severe trouble”.

The aims were 1) to assess the prevalence of perceived symptoms within the previous week in 12 anatomic regions (Figure 1); 2) to assess the prevalence of clinical findings in nine anatomic regions in the neck, back, and upper extremities (Table 1); and 3) to investigate the co-existence of perceived musculoskeletal symptoms (soreness, pain, or discomfort) and clinical musculoskeletal findings found using simple, standardised, clinical tests such as range of motions, functional assessment, resistance tests and pain-rating.

**Table 1:** The clinical examination

	**Palpation soreness/ pain**	**Trigger points**	**Active range of motion**	**Functional (active) motion**	**Pain at active motion**	**Muscle test (resistance test) for force and pain**	**Nerve roots or peripheral nerves in tests**	**Hypermobile**
***Neck***								
Flexion			×~		×			
Extension			×~		×			
Rotation			×~		×			
Lateral flexion			×~		×			
Foramen compression							×C5-Th1	
Posterior paravertebral neck muscles	×	×						
Scalenus muscles	×	×						
***Upper back***								
Trapezius muscles	×	×						
Supraspinatus muscles	×	×						
Infraspinatus muscles	×	×						
Levator scapulae muscles	×	×						
***Lower back***								
Finger-floor-distance				×~				×+
***Shoulders***								
Flexion			× ~ *		× ~ *			
Abduction			× ~ *		× ~ *	×~	× Ax Ss	
Extension			×~		×~			
Cross over test				× ~ *	× ~ *			
Functional external rotation				×*				
Functional internal rotation				×*				
***Elbows***								
Flexion			×~		×	×~	× Mc	
Extension			×~		×	×~	× Ra	×+
Supination			×~		×	×~	× Mc Ra	
Pronation			×~		×	×~	× Me	
Midposition						×		
Lateral epicondyle	×~							
Medial epicondyle	×~							
Wrist flexors, muscle bellies & tendons	×~							
Wrist extensors, muscle bell. & tendons	×~							
***Wrists and hands***								
Extension of the wrist			×~		×	×~	× Ra	
Flexion of the wrist			×~		×	×~	× Me Ul	
1^st^ tunnel (ext. poll. br., abductor long.)	×~							
Finkeltstein	×~							
Tinnel’s test							× Me	
Opposition of 1^st^ finger				×~	×		× Me	
Abduction of 5^th^ finger				×~			× Ul	
Froment’s test				×			× Ul	
Extension of little finger								×+
Thumb to forearm								×+
***Knees***								
Knees								×+

Symbol key:

× = Examined.

~ = As described by Stanley Hoppenfeld.

* = From the Constant-Murley shoulder test.

+ = For Beighton’s hypermobility score.

C5-Th1 = Nerve root C5-Th1.

Ax = axillary nerve.

Ss = Subscapular nerve.

Ra = radial nerve.

Me = median nerve.

Mc = musculocutaneous nerve.

## Methods

### Design

The present study was held as an explorative, cross-sectional study of perceived musculoskeletal symptoms and clinical musculoskeletal findings in professional symphony orchestra musicians in Denmark. The perceived problems were assessed by a self-reported rating scheme containing a numeric and a verbal rating scale for 12 anatomic regions (Figure 1); this scheme was completed by each participant prior to the clinical examination and was blinded to the examiner. The clinical findings were achieved through a structured clinical examination of the neck, back and upper extremities, which was constructed for this study (Table 1).

### Population

All musicians employed for playing in six professional symphony orchestras in Denmark were asked to participate in the clinical examination. In total 441 musicians were asked of which the half (49%) volunteered. Absence excluded participation in the examination. There were no further exclusion criteria.

### Setting

Each orchestra was visited in two steps: First, an oral information meeting about the study was held followed by distribution of written information; secondly, for performing the clinical examinations. All clinical examinations were performed by the same two examiners, a medical doctor and a physiotherapist, and were performed in available examination rooms at the musicians’ workplace, the concert houses. The musicians who signed up for the examination were divided randomly between the two examiners without information of the musicians’ age or instrument group.

Although always in relation to an orchestral rehearsal it was arranged that the clinical examination took place at different hours in the different orchestras: In one orchestra the musicians were examined before the orchestral rehearsal; in two the musicians had permission to leave the orchestral rehearsal to be examined also during the rehearsal; in three the musicians were only examined in the scheduled breaks during a work-day meaning in formalised breaks and right before and after rehearsal (see Table 2). Data were collected from March to October 2007. However, no data were collected during July and August due to the orchestras’ summer vacation.

**Table 2:** Participation according to examination time

	***Employees***	***Participants***	
	**N**	**% (N)**	**95% ci**
**Examined during rehearsal:**			
Orchestra no. 1	68	67.6% (46)	56.4-78.9%
Orchestra no. 5	57	82.5% (47)	72.5-92.4%
**During rehearsals, in total**	**125**	**74.4% (93)**	**66.7-82.1%**
**Examined in formalized breaks:**			
Orchestra no. 2	78	44.9% (35)	33.7-56.0%
Orchestra no. 4	68	42.6% (29)	30.8-54.5%
Orchestra no. 6	103	33.0% (34)	23.9-42.2%
**In formalized breaks, in total**	**249**	**39.4% (98)**	**33.3-45.5%**
**Examined before rehearsal:**			
Orchestra no. 3	67	37.3% (25)	25.6-49.0%
**Before rehearsals, in total**	**67**	**37.3% (25)**	**25.6-49.0%**
*In total*	*441*	*49.0%* (*216*)	*44.3-53.7%*

### Scoring of perceived symptoms

The data on the musicians’ perceived symptoms were obtained through a self-reported rating scheme of maximally perceived musculoskeletal trouble (ache, pain, or discomfort) within the previous seven days. The questions applied to any perceived musculoskeletal trouble - whatever had caused it - in 12 anatomic sites. The musculoskeletal trouble was rated on a 10 point numeric and verbal scale chosen according to the Borg category ratio scale system [24,25]. The questions were asked in line with the Nordic Musculoskeletal Questionnaire [26,27]. The rating scheme was filled in shortly before the clinical examination and was handed in, blinded to the examiner, at the start of the examination.

### The clinical examination

The clinical examination was constructed for the purpose. The aim was to develop a non-expensive instrument, easy to use in any clinical setting without specialised tools, and informative regarding signs indicating an impaired physical functional level of importance from using the musical instruments. This was addressed by simple clinical standard measures including range of motion, resistance tests, neurological tests, soft tissue palpation and pain scores; additionally current hypermobility was tested according to the Beighton score [28]. Anatomically the examination focused on the neck, back, and upper extremities (see Table 1).

Active motion was tested as active range of motion in the neck (six directions) and in the shoulders (three directions) and as composite functional motion in the shoulders and in the fingers. In case of impaired range of motion the test was repeated three times and only the best result was registered. In case of impaired active range of motion or pain at active range of motion in the shoulder, passive range of motion and pain at passive range of motion were tested subsequently. Resistance tests were performed to check for symmetrically, equally good force and both impaired performance and pain were registered. Palpation of specific muscle sites in the neck and upper back was performed using a flat thumb pressure of 3–4 kg/cm^2^ which was regularly checked with a pressure algometer [29]. Pain and palpation soreness was throughout the examination noted on a four level scale: None, mild, moderate, severe. None or mild was solely reported verbally by the examined musician, moderate pain or soreness was a combination of jump response and a verbal confirmation of the pain/soreness level, and severe pain was a combination of jump sign and a verbal confirmation of the pain/soreness level. Jump response was defined as a brief local or general contraction of muscle, and jump sign was defined as involuntary withdrawing from palpation or wincing, most often together with a pain-related vocalization [30,31]. Hypermobility was tested according to the Beighton score [28]. Opposition of the thumb was measured to the distal palmar crease [32]. All other examinations of active and passive motion were performed according to the description by Stanley Hoppenfeld and/or the Constant-Murley shoulder score [33,34]. These tests were chosen as they are well-defined, easy to perform, and either commonly used or similar to tests that are commonly used for examining outpatients in physical therapy as well as in occupational medicine.

### Variables

The biological variables “gender” and “age” and the work-related variables “mastered main instrument” and “orchestra of employment” were known as baseline data for all musicians in the study population (see Table 3). Based on their main instrument the musicians were divided into five groups of which four were traditional and the fifth a smaller group named “others”: High strings = violin, viola; low strings = cello, double bass; woodwinds = flute, oboe, clarinet, bassoon; brass instruments = horn, trumpet, trombone, tuba; others = timpani and percussion, harp, piano and organ.

**Table 3:** Study population, representativity of participants, and distribution of examined participants between examiners

	**Study population N =** **441**	**Participants N = 216**	**Confidence interval for participants**	**Participants examined by examiner1**	**Participants examined by examiner2**
	**% (N)**	**% (N)**	**95% ci**	**N = 90**	**N = 126**
**Gender**					
Men	61.0% (269)	54.2% (117)	47.5-60.8%	44%	56%
Women	39.0% (172)	45.8% (99)	39.2-52.5%	39%	61%
*In total*	*100.0% (441)*	*100.0% (216)*	-	*42%*	*58%*
**Age groups**					
20-29	7.3% (32)	5.1% (11)	2.1-8.0%	45%	55%
30-39	33.1% (146)	32.9% (71)	26.6-39.2%	44%	56%
40-49	23.6% (104)	27.3% (59)	21.3-33.3%	39%	61%
50-59	22.7% (100)	24.5% (33)	18.8-30.3%	42%	58%
60-69	13.2% (58)	10.2% (22)	6.1-14.2%	41%	59%
70-79	0.2% (1)	0% (0)	-	0%	0%
*In total*	*100.1% (441)*	*100.0% (216)*	**-**	*42%*	*58%*
**Instrument groups**					
High strings	44.2% (195)	44.4% (96)	37.8-51.1%	40%	60%
Low strings	16.8% (74)	18.5% (40)	13.3-23.7%	43%	58%
Brass players	16.8% (74)	14.4% (31)	9.7-19.1%	42%	58%
Woodwinds	15.6% (69)	15.7% (34)	10.9-20.6%	38%	62%
Others (percussion, harp, keyboard)	6.6% (29)	6.9% (15)	3.5-10.45	60%	40%
*In total*	*100.0% (441)*	*99.9% (216)*	-	*42%*	*58%*
**Orchestra**					
No. 1	15.4% (68)	21.3% (46)	15.8-26.8%	50%	50%
No. 2	17.7% (78)	16.2% (35)	11.3-21.1%	54%	46%
No. 3	15.2% (67)	11.6% (25)	7.3-15.9%	48%	52%
No. 4	15.4% (68)	13.4% (29)	8.9-18.0%	41%	59%
No. 5	12.9% (57)	21.8% (47)	16.2-27.3%	47%	53%
No. 6	23.4% (103)	15.7% (34)	10.9-20.6%	6%	94%
*In total*	*100%* (*441)*	*100%* (*216)*	-	*42%*	*58%*

Data on the degree of perceived problems within the previous week were obtained from the self-reported rating scheme where the musicians for all anatomic regions rated their problems on a 10 point scale (Figure 1). This scale was later converted into a four-point scale with the following cut points: 0 = no trouble, 1–3 = mild, 4–6 = moderate, and 7–9 = severe problems. Cut points were chosen on the basis of verbal accordance between the detailed 10 point verbal rating scale on the self-reported rating scheme and the 4 point verbal scale in the clinical examination.

For all clinical tests negative as well as positive clinical findings were registered at the time of examination; the references may be conferred for details on the criteria for positive findings [29,32-34].

Binary variables were calculated for perceived symptoms, for clinical findings, and for co-existence of perceived symptoms and clinical findings for each anatomic region (see Table 4 and Figure 2). For perceived symptoms these were constructed as “none” or “any trouble” (Table 1 and Figure 2), as “none or mild” versus “moderate or severe” (Figure 2), and as “severe” or “less than severe” (Figure 2), as used for the sensitivity test. The binary variable for clinical findings in one anatomic region is a summed variable of all separate tests performed in the particular anatomic region as described in Table 1; 0 indicating all tests were negative, 1 that 1 or more tests were positive. The result of this summed variable is shown in Table 4 and is used for sensitivity and specificity calculations.

**Table 4:** **Over-all examination results by gender and by instrument group**

	**In total**	**Men**	**Women**	**High strings**	**Low strings**	**Woodwinds**	**Brass players**	Others*
	**N = 216**	**N = 117**	**N = 99**	**N = 96**	**N = 40**	**N = 31**	**N = 34**	**N = 15**
**Neck**								
P	64.8% (140)	57.3% (67)	73.7% (73)	70.8% (68)	67.5% (27)	54.8% (17)	58.8% (20)	53.3% (8)
C	76.4% (165)	72.7% (85)	80.8% (80)	75.0% (72)	80.0% (32)	77.4% (24)	76.5% (26)	73.3% (11)
P & C	53.7% (116)	47.9% (56)	60.6% (60)	57.3% (55)	55.0% (22)	51.6% (16)	50.0% (17)	40.0% (6)
**Upper back**								
P	53.2% (115)	42.7% (50)	65.7% (65)	57.3% (55)	52.5% (21)	51.6% (16)	47.1% (16)	46.7% (7)
C	67.6% (146)	58.1% (68)	78.8% (78)	72.9% (70)	70.0% (28)	58.1% (18)	64.7% (22)	53.3% (8)
P & C	39.8% (86)	28.2% (33)	53.5% (53)	45.8% (44)	40.0% (16)	35.5% (11)	35.3% (12)	20.0% (3)
**Lower back**								
P	50.5% (109)	49.6% (58)	51.5% (51)	50.0% (48)	50.0% (20)	51.6% (16)	52.9% (18)	47.7% (7)
C	6.0% (13)	9.4% (11)	2.0% (2)	3.1% (3)	10.0% (4)	6.5% (2)	11.8% (4)	0.0% (0)
P & C	0.5% (1)	6.8% (8)	2.0% (2)	3.1% (3)	5.0% (2)	3.2% (1)	11.8% (4)	0.0% (0)
**Left shoulder**								
P	52.8% (114)	47.0% (55)	59.6% (59)	60.4% (58)	55.0% (22)	38.7% (12)	47.1% (16)	40.0% (6)
C	50.9% (110)	54.7% (64)	46.5% (46)	53.1% (51)	52.5% (21)	35.5% (11)	61.8% (21)	40.0% (6)
P & C	30.6% (66)	29.1% (34)	32.3% (32)	34.4% (33)	27.5% (11)	16.1% (5)	38.2% (13)	26.7% (4)
**Right shoulder**								
P	50.0% (108)	46.2% (54)	54.5% (54)	46.9% (45)	60.0% (24)	48.4% (15)	50.0% (17)	46.7% (7)
C	64.8% (140)	71.8% (84)	56.6% (56)	64.6% (62)	60.0% (24)	71.0% (22)	70.6% (24)	53.3% (8)
P & C	34.3% (74)	33.3% (39)	35.4% (35)	33.3% (32)	37.5% (15)	32.3% (10)	35.3% (12)	33.3% (5)
**Left elbow**								
P	24.5% (53)	23.1% (27)	26.3% (26)	22.9% (22)	35.0% (14)	19.4% (6)	20.6% (7)	26.7% (4)
C	31.9% (69)	23.9% (28)	37.4% (37)	35.4% (34)	25.0% (10)	29.0% (9)	26.5% (9)	46.7% (7)
P & C	11.6% (25)	11.1% (13)	10.1% (10)	12.5% (12)	15.0% (6)	3.2% (1)	11.8% (4)	13.3% (2)
**Right elbow**								
P	19.4% (42)	17.9% (21)	21.2% (21)	20.8% (20)	30.0% (12)	19.4% (6)	5.9% (2)	13.3% (2)
C	31.9% (69)	23.9% (28)	34.4% (34)	40.6% (39)	25.0% (10)	32.3% (10)	17.7% (6)	26.7% (4)
P & C	10.7% (23)	9.4% (11)	9.1% (9)	10.4% (10)	17.5% (7)	9.7% (3)	5.9% (2)	6.7% (1)
**Left hand/wrist**								
P	36.1% (78)	32.5% (38)	40.4% (40)	36.5% (35)	37.5% (15)	35.5% (11)	29.4% (10)	46.7% (7)
C	31.5% (68)	28.2% (33)	35.4% (35)	32.3% (31)	27.5% (11)	35.5% (11)	29.4% (10)	33.3% (5)
P & C	15.3% (33)	13.7% (16)	17.2% (17)	15.6% (15)	12.5% (5)	16.1% (5)	17.7% (6)	13.3% (2)
**Right hand/wrist**								
P	29.6% (64)	27.4% (32)	32.3% (32)	27.1% (26)	40.0% (16)	35.5% (11)	14.7% (5)	40.0% (6)
C	37.5% (81)	37.6% (44)	37.4% (37)	39.6% (38)	27.5% (11)	38.7% (12)	38.2% (13)	46.7% (7)
P & C	14.4% (31)	17.1% (20)	11.1% (11)	9.4% (9)	20.0% (8)	22.6% (7)	5.9% (2)	33.3% (5)
**Hips**								
P	18.5% (40)	19.7% (23)	17.2% (17)	18.8% (18)	25.0% (10)	16.1% (5)	14.7% (5)	13.3% (2)
**Knees**								
P	30.1% (65)	27.4% (32)	33.3% (33)	29.2% (28)	30.0% (12)	32.3% (10)	35.3% (12)	20.0% (3)
**Ankles & feet**								
P	22.7% (49)	18.8% (22)	27.3% (27)	22.9% (22)	22.5% (9)	25.8% (8)	20.6% (7)	20.0% (3)

*others: Percussion, harp, keyboard.

P = perceived symptoms; the prevalence rates of any reported self-experienced musculoskeletal trouble within the previous week. C = clinical findings; the summed variable of clinical findings in each anatomic region. P & C = perceived symptoms and clinical findings; the musicians who had perceived symptoms as well as clinical findings.

### Missing data

There were no missing data in the baseline data. Regarding perceived trouble, there were no missing data in 10 anatomic categories and just 1/216 (0,005%) for the last 2 categories. In the clinical examination maximally 2 observations were missing in any measurement. This due to a few participants who, due to their medical condition (pregnancy or previous surgery), were not able to perform very specific parts of the clinical examination.

### Statistical methods

Crude prevalence rates and proportion rates with 95% confidence intervals were calculated. Groups were compared using a chi^2^ test and the level of significance was defined as p < 0.05. The difference between the prevalence rates of perceived symptoms and the prevalence rates of clinical findings were simply calculated as percentage points expressed as the numerical value:

(1) $$\left|\%\;\text{with symptoms}–\%\;\text{with clinical findings}\right|$$

As perceived symptoms in this study were defined as the true health outcome, perceived symptoms served as the true standard measure for sensitivity and specificity calculations:

(2) $$\text{Sensitivity=}\;\frac{\text{number of true positives}}{\left(\text{number of true positives+number of false negatives}\right)}\times 100\%$$

(3) $$\text{Specificity=}\;\frac{\text{number of true negatives}}{\left(\text{number of true negatives+number of false positives}\right)}\times 100\%$$

True positive: perceived symptoms and clinical findings.

False positive: no perceived symptoms but clinical findings.

True negative: no perceived symptoms and no clinical findings.

False negative: perceived symptoms but no clinical findings.

To ensure protection of anonymity and to minimize coincidental results from analyses on a case level the groups used for analyses were not smaller than gender (men, women), instrument groups (high strings, low strings, woodwinds, brass winds, others), and the total group. Data management was conducted using Stata SE 10.0 (StataCorp LP, College Station, Texas, US).

### Ethics

The study was carried out in compliance with the Helsinki declaration; it was registered at the Danish Data Protection Agency (journal no. 2006-41-7194) and approved by the Research Ethics Committee (project ID VF-20060086) and was conducted with written consent obtained from all participants. Participation was voluntarily and personal information was kept anonymous to 3^rd^ party.

## Results

### Participation

The musicians’ participation in the clinical examination varied between orchestras as listed in Table 2. The two orchestras where the musicians were permitted free access to the clinical examination during working-hours had significantly higher participation rates than the orchestras where the musicians were only offered to be examined during formalized breaks or before rehearsal. There was no statistically significant difference in the participation between musicians only being permitted to be examined in breaks or before rehearsals.

Participation by gender, age groups, instrument groups, and orchestra of employment are all listed as proportion rates in Table 3. Regarding the two biological variables”gender” and “age”, the clinically examined participants were representative of the study population according to the percentage of participants and the corresponding 95% confidence intervals; additionally assessed by a chi^2^ test there were no statistically significant difference between the study population and the participants regarding gender and age groups. Likewise, the participants were representative for the study population regarding instrument groups and without statistically significant difference between the two groups. However, at the orchestra level the clinically examined half of the musicians was statistically significant different from the non-examined half of the study population. Tested one to one this difference was due to participants from Orchestra no. 5 being overrepresented and from Orchestra no. 6 being underrepresented compared to the participation of musicians from the other orchestras (Table 3).

Although the musicians were divided randomly between the examiners there turned out to be a tendency of a 40:60 distribution ratio regarding gender, instrument and age groups, see Table 3.

### Prevalence of symptoms and findings

According to the aim, clinical findings were assessed in nine anatomic regions. Only the summed variables of clinical findings in each anatomic region were used for analysing the sensitivity of the test. Prevalence rates of perceived symptoms within the last seven days, prevalence rates of summed clinical findings, and prevalence rates of true positive findings - being those who reported perceived symptoms and also had clinical findings - are all shown in Table 4. The prevalence rates of perceived symptoms were highest in the neck, upper and lower back and shoulders. The prevalence rates of clinical findings and of co-existing symptoms and findings were both highest in the area comprising the neck, shoulders and upper back. Generalised hypermobility was rare and affected only seven women and no men.

Comparing the prevalence rates of any perceived symptoms (whatever the cause) and clinical findings (of which the clinical examination for the lumbar back solely comprised the finger-to-floor distance) the results for the lower back stood out with a remarkably huge difference between perceived symptoms and objective findings, clinical findings being 40.0 to 49.5 percentage points (pp) lower than the prevalence of self-reported problems in all groups of musicians. Regarding the other regions the variation in the differences between the prevalence of self-reported symptoms and of clinical findings were smaller ranging, expressed as numerical values, within 4.2 - 22.6 pp for the neck, 6.5 - 17.6 pp for the upper back, 0–14.7 pp for the left shoulder and 0–25.6 pp for the right, 0.8 - 20.0 pp for the left elbow and 5.0 - 19.8 pp for the right, 0–13.4 pp for the left hand and wrist, and from 3.2 - 23.5 pp for the right hand and wrist. Comparing these pp-measures to Table 1 is seen that the discrepancy between the prevalence rates of symptoms and clinical findings was not just narrowing or widening depending on the number of tests.

### Sensitivity and specificity

The low co-existence of perceived musculoskeletal trouble and present clinical findings naturally draws attention towards the sensitivity and specificity of the clinical examination. As seen in Table 5 differences in sensitivity and specificity were seen between instrument groups and between genders in all anatomic regions. The sensitivity was highest for the neck, shoulders, and upper back.

**Table 5:** **Sensitivity and specificity by instrument group and by gender for all anatomic regions**

	**Neck**	**Upper back**	**Lower back**	**Left shoulder**	**Right shoulder**	**Left elbow**	**Right elbow**	**Left hand & wrist**	**Right hand & wrist**
***By instrument***									
**High strings**									
Sensitivity	81%	80%	6%	57%	71%	55%	50%	43%	35%
Specificity	65%	37%	100%	53%	41%	70%	61%	74%	56%
**Low strings**									
Sensitivity	81%	76%	10%	50%	63%	43%	58%	33%	50%
Specificity	23%	37%	90%	44%	44%	84%	89%	76%	88%
**Woodwinds**									
Sensitivity	94%	69%	6%	42%	66%	17%	50%	45%	64%
Specificity	43%	53%	93%	45%	25%	68%	72%	70%	75%
**Brass players**									
Sensitivity	85%	75%	22%	81%	71%	57%	100%	60%	40%
Specificity	36%	44%	100%	56%	29%	81%	88%	83%	62%
Others*									
Sensitivity	75%	43%	0%	67%	71%	50%	50%	29%	83%
Specificity	29%	75%	100%	78%	63%	55%	77%	63%	78%
***By gender***									
**Men**									
Sensitivity	84%	66%	14%	62%	72%	48%	52%	42%	63%
Specificity	42%	48%	95%	52%	29%	83%	82%	78%	72%
**Women**									
Sensitivity	82%	82%	4%	54%	65%	38%	43%	43%	34%
Specificity	23%	26%	100%	65%	53%	63%	68%	69%	61%
***In total***									
Sensitivity	83%	75%	9%	58%	69%	47%	55%	42%	48%
Specificity	36%	41%	97%	57%	39%	73%	73%	75%	67%

*Others: Percussion, harp, keyboard.

The sensitivity and specificity in Table 5 were calculated on the basis of the binary measure of absence or presence of any perceived problem. Keeping perceived symptoms as the true standard, sensitivity calculations were repeated increasing the degree of perceived symptoms. As illustrated in Figure 2 increasing the degree of perceived symptoms slightly increased the sensitivity of the clinical examination, mostly for the upper extremities, whereas the specificity remained almost unchanged.

## Discussion

### Main findings

Accessibility to be examined appeared to be of major importance for a high participation rate; among musicians who had permission to be examined at any time during a rehearsal about twice as many took part compared to the musicians who only had access to be examined before rehearsals or in breaks.

Self-reported subjective symptoms and clinical findings were most common in the neck, back, and shoulders. The rather small co-existence between self-reported symptoms and clinical findings was reflected in the sensitivity and specificity of the examination which ultimately indicates, that a clinical screening examination as used in this study cannot be used as a stand-alone diagnostic test.

### Interpretation of findings

Accessibility to be examined was demonstrated to be of major importance for a high participation rate. Among the musicians who had permission to be examined at any time during a rehearsal 74.4% (95% ci: 66.7-82.1%) participated, which was almost twice as much as among the musicians who only had access before rehearsals or in breaks.

The constructed clinical examination was – as planned - a non-expensive instrument, easy to perform due to the use of well-known, standardised tests [25,26,29,32-34]. Furthermore it was easy to use in the quite primitive clinical settings without specialised tools. However, the test was not as informative as was the aim. In particular, the test was not diagnostically informative.

Perceived symptoms within the previous week as well as present clinical findings were assessed in compliance with the study aims. Self-reported subjective symptoms were most common in the neck, upper and lower back, and shoulders while less prevalent in the distal upper limbs (elbows, hands and wrists) and lower limbs (hips, knees, ankles/feet). Clinical findings were most common in the neck, upper back, and shoulders, and as for perceived symptoms also clinical findings were generally less frequent in the elbows, hands and wrists. Eventually clinical findings were very low in the lumbar back which was, however, only tested by measuring the finger-floor distance.

Generally, the co-existence of symptoms and findings was quite poor, and this clinical examination could not be used as a screening method to identify musicians experiencing musculoskeletal symptoms as the high sensitivity needed to do this was not achieved. However, as shown in Figure 2, a slight increase in sensitivity was demonstrated when the degree of perceived problems was higher. If the clinical examination in this study had made it possible to identify those musicians who reported musculoskeletal symptoms, the examination could have been tested as a general screening test to point out musicians showing symptoms of e.g. RSI already at quite early stages as musculoskeletal disorders among musicians often tend to be RSI of which early stages often are symptoms like soreness or pain, and RSI also include non-specific disorders [17,18]. The insufficient sensitivity of an examination could possibly be compensated by a high specificity making the examination a good screening method to at least exclude the presence of disorders in case of lack of clinical findings. Yet in this study this was only the case for the lower back, which was only roughly tested by assessing the finger-to-floor distance. Therefore this examination cannot, whatsoever, be used as a stand-alone diagnostic screening test but leaves the musicians’ symptoms to the old diagnostic challenge of clinical diagnostic depending on the individual examiner’s clinical knowledge and experience of diagnosing based on the entire picture of subjective and clinical findings.

### Considerations on the examination

The construction of the examination was intended to comprise the overall elements of what a musician presenting a symptom as ache, pain or discomfort could be expected to undergo at a typical first consultation at different kinds of health care practitioners such as the doctor in occupational medicine or the physiotherapist. Therefore the chosen tests were not only easy to perform; they were also common in the examination of outpatients in physical therapy as well as in occupational medicine. Furthermore all elements of the examination were well-defined and previously standardised [32-34]. However, some weaknesses should be given attention. For instance, that the examination only gave answer to whether there were symptoms and/or clinical findings, but not whether they interfered with playing the instrument. There was also a lack of information about any knowledge about current diagnoses, medical treatment of current problems, and the exposure prior to the examination. Furthermore inter-investigator analysis between the two examiners, a medical doctor and a physiotherapist, was not made.

### Generalizability and limitations

The participants in this study were representative of the study population by gender, age, and instrument groups. As symphony orchestras internationally are very similar regarding instrumentation, hierarchical organization and with instruments played in the same way, the sample of musicians in this study could be expected to be representative for a large part of symphony orchestra musicians worldwide [35,36]. However, there were several possible biases in this study among which the low participation rate (49%) must be mentioned. As the results only account for the half of the musicians in this study, these results can only be indicative of high frequencies of symptoms and clinical findings - as well as of a low co-existence of symptoms and findings - among orchestra musicians. Another factor that may influence on the results emphasizing that the results in this explorative study are only indicative was the lack of truly objective or more reproducible measures e.g. the estimation of bilaterally equal good muscle strength not being determined by the use of a dynanometer but only by simple manual muscle testing. Among other possible biases was the lack of exposure information, not only the currently as well as the cumulated exposure time, but also taking into account that differences in the currently played musical repertoire may result in differences in the current ergonomic strain exposure and thus differences in the musicians’ symptoms and findings. Furthermore elements from the psychosocial working environment may influence on the musicians’ musculoskeletal problems [37]. And although on an even higher level the local labour market conditions – such as working hours and sickness compensation – as well as the local health care system and accessibility to health care services may lead to different results between countries.

### Considerations of studies of a work-attending population

Studies in different work-attending occupational groups are common, and perceived symptoms are widely investigated using the standardized Nordic Musculoskeletal Questionnaire (NMQ) or adaptations hereof [38-41]. The NMQ does not operate on the diagnostic level but is concerned with the respondents’ subjective experience of presence and extent of possible health problems [26]. This makes sense as the experienced extent of a health problem is very individual and as the studied population is work-attending and therefore must be expected to be in relatively good health, and as application of clinical diagnoses in a work-attending group can be seen as sickening a basically healthy group. Considering work-attending individuals as basically healthy is also consistent with the usually low prevalence of actual, clinical diagnoses in such studies [19-23]. Furthermore it should be taken into account that musculoskeletal symptoms fluctuate by nature. This contributes to the difficulty in achieving an exact depiction of a musculoskeletal problem solely through a clinical examination. Finally it should also be kept in mind, that even if extending the examination from diagnoses to simple clinical findings – as in this study -, the measurement units used for perceived symptoms and for clinical findings are different, as symptoms usually are described as a period prevalence (such as week prevalence or year prevalence as in the NMQ) and clinical findings as a point prevalence. So although the research design itself (combining self-reported data on perceived problems and a clinical examination) is very traditional, it might not be truly suitable, as bias would most likely be inherent in the very design. The mismatch between perceived symptoms and clinical findings may, of course, to some extent be addressed as study specific information bias e.g. recall bias or increased symptom reporting due to the attention, but it may also be due to a mismatch between the measuring instruments, namely a period measuring questionnaire as the NMQ mainly constructed as a screening instrument for harmful workplaces and a solely point prevalent clinical examination. Furthermore the clinical examination is characterized by crude examination measures that are usually used to test patients for objective signs of dysfunction. These measures may be more suitable and informative when used among employees who cannot perform at their regular full-time capacity than in a work-attending group. Thus there may also be a mismatch between what is measured in this study and in whom, namely sickness measures in generally healthy people, although maybe at risk.

### Considerations about future research

Taking into account how the participation rates differed according to the symphony orchestra musicians’ access to be examined, as shown in Table 2, future studies should preferably be designed so that participants have free access to be examined during the workday. The purely cross-sectional design could be kept for baseline or follow-up prevalence measuring or hypothesis generation. If so, and repeating this study, optimizing the present clinical examination must be recommended: The current study examined the active range of motion and pain of motion in the neck and shoulders, however, this did not reveal anything about the actual and playing-related level of function. More functional tests, possibly of playing-related relevance, could be a benefit, just as the informative level of the clinical examination may benefit from using measuring instruments such as inclinometers, dynanometers, and algometers. Also self-reported information on whether perceived symptoms impact functionally could preferably be added. For case-defining studies focusing at the transition from healthy to sick momentary assessment tools may be preferred examining participants selected on basis of present problems, and not only on basis of problems reported within a period.

## Conclusions

By using the self-reported rating scheme (Figure 1) and the clinical examination (Table 1), both designed on the basis of standardised tests [24-27]; [33,34], the one-week period prevalence of perceived symptoms, the point prevalence of clinical findings, and the co-existence of perceived symptoms and clinical findings could be assessed in a work-attending group of professional symphony orchestra musicians. The highest prevalence proportions of perceived symptoms within the previous week were in the neck, back, and shoulders. Very consistently with the presence of any perceived symptoms, the prevalence rate of any clinical findings were highest in the neck, upper back, and shoulders and also the co-existence of symptoms and findings was highest in those regions. Regarding clinically objective identification of musicians experiencing subjective problems this was not possible or just coincidental, as the sensitivity of the test was too low for this and differed between the anatomic regions. Thus this clinical examination should not be considered being a diagnostic stand-alone test (Figure 2).

Musculoskeletal symptoms fluctuate. But as perceived symptoms usually are measured as period prevalence rates while clinical musculoskeletal examinations usually are point prevalence rates these measuring methods thus are different and not directly comparable but supplemental measures. Other research methods should be considered when studying musculoskeletal problems in occupational groups although the combination of self-reported symptoms and a clinical examination is a well-known research tradition which can be informative as a baseline study or in other studies where information about prevalence rates is desired.

## Competing interests

The authors have no financial or non-financial conflicts of interest regarding the present study.

## Authors' contributions

In alphabetical order: Conception and design of the study: HMP, JB, NW. Data collection: HMP. Data analysis: HMP, NW. Interpretation of data: CM, HMP, JB, JWH, NW. Drafting the article: HMP, NW. Revising the article critically: CM, HMP, JB, JWH, NW. All authors read and approved the final manuscript. Guarantor of the paper: HMP, NW.

## References

[B1] Nyman T Wiktorin C Mulder M Johansson YL Work postures and neck–shoulder pain among orchestra musicians Am J Ind Med 2007 50 370 376 17427201 10.1002/ajim.20454

[B2] Foxman I Burgel BJ Musicians health and safety: Preventing playing-related musculoskeletal disorders musculoskeletal disorders AAOHN J 2006 54 309 316 16862878 10.1177/216507990605400703

[B3] Turner-Stokes L Reid K Three-dimensional motionanalysis of upper limb movement in the bowing arm of string-playing musicians Clin Biomech 1999 14 426 433 10.1016/s0268-0033(98)00110-7 10521625

[B4] Edling CW Fjellman-Wiklund A Musculoskeletal disorders and assymetric playing postures of the upper extremity and back in music teachers Medical Probl Perform Artists 2009 24 113 118

[B5] Norris RN Applied ergonomics: adaptive equipment and instrument modification for musicians Md Med J 1993 42 271 275 8350686

[B6] Bernard BP Musculoskeletal disorders and workplace factors NIOSH 1997 97:141 http://www.cdc.gov/niosh/docs/97-141/pdfs/97-141.pd

[B7] Larson B Søgaard K Rosendal L Work related neck-shoulder pain: a review on magnitude, risk factors, biochemical characteristics, clinical picture and preventive interventions Best Pract Res Clin Rheumatol 2007 21 447 463 17602993 10.1016/j.berh.2007.02.015

[B8] Keyserling WM Workplace risk factors and occupational musculoskeletal disorders, Part 2: A review of biomechanical and psychophysical research on risk factors associated with upper extremity disorders Am Ind Hyg Assoc J 2006 61 231 243 10.1080/15298660008984532 10782195

[B9] Kaufman-Cohen Y Ratzon NZ Correlation between risk factors and musculoskeletal disorders among classical musicians Occup Med 2011 61 90 95 10.1093/occmed/kqq196 21273187

[B10] Bejjani FJ Kaye GM Benham M Musculoskeletal and neuromuscular conditions of instrumental musicians Arch Phys Med Rehabil 1996 77 406 413 8607768 10.1016/s0003-9993(96)90093-3

[B11] Zaza C Playing-related musculoskeletal disorders in musicians: A systematic review of incidence and prevalence CMAJ 1998 158 1019 1025 9580730 PMC1229223

[B12] Bragge P Bialocerkowski A McMeeken J A systematic review of prevalence and risk factors associated with playing-related musculoskeletal disorders in pianists Occup Med 2006 56 28 38 10.1093/occmed/kqi177 16275655

[B13] Leaver R Harris EC Palmer KT Musculoskeletal pain in elite professional musicians from British symphony orchestras Occup Med 2011 61 549 555 10.1093/occmed/kqr129 PMC3428866 22003061

[B14] Paarup HM Baelum J Holm JW Manniche C Wedderkopp N Prevalence and consequences of musculoskeletal symptoms in symphony orchestra musicians vary by gender: a cross-sectional study BMC Musculoskelet Disord 2011 12 223 21978278 10.1186/1471-2474-12-223 PMC3221643

[B15] Hoppmann RA Instrumental musicians’ hazards Occup Med 2001 16 619 631 11567922

[B16] Lederman R Neuromuscular and musculoskeletal problems in instrumental musicians Muscle Nerve 2003 27 549 561 12707974 10.1002/mus.10380

[B17] Yassi A Repetitive strain injuries Lancet 1997 349 943 947 9093264 10.1016/S0140-6736(96)07221-2

[B18] Fry HJH The treatment of overuse syndrome in musicians. Results in 175 patients J R Soc Med 1988 81 572 575 3184089 10.1177/014107688808101007 PMC1291799

[B19] Stål M Moritz U Johnsson B Pinzke S The natural course of musculoskeletal symptoms and clinical findings in upper extremities of female milkers Int J Occup Environ Health 1997 3 190 197 9891118 10.1179/oeh.1997.3.3.190

[B20] Åkeson I Johnsson B Rylander L Moritz U Musculoskeletal disorders among female dental personnal – clinical examination and a 5-year follow-up study of symptoms Int Arch Occup Environ Health 1999 72 395 403 10473839 10.1007/s004200050391

[B21] Gerr F Marcus M Ensor C Kleinbaum D Cohen S Edwards A Gentry E Ortiz DJ Monteilh C A prospective study of computer users: I. Study design and incidence of musculoskeletal symptoms and disorders Am J Ind Med 2002 41 221 235 11920966 10.1002/ajim.10066

[B22] Andersen JH Kaergaard A Mikkelsen S Jensen UF Frost P Bonde JP Fallentin N Thomsen JF Risk factors in the onset of neck/shoulder pain in aprospective study of workers in industrial and service companies Occup Environ Med 2003 60 649 654 12937185 10.1136/oem.60.9.649 PMC1740607

[B23] Kryger AI Andersen JH Lassen CF Brandt LP Vilstrup I Overgaard E Thomsen JF Mikkelsen S Does computer use pose an occupational hazard for forearm pain; from the NUDATA study Occup Environ Med 2003 60 e14 http://www.occenvmed.com/cgi/content/full/60/11/e14 14573725 10.1136/oem.60.11.e14 PMC1740406

[B24] Borg G Borg E A new generation of scaling methods: Level-anchored ratio scaling Psychologica 2001 28 15 45

[B25] Neely G Ljunggren G Sylvén C Borg G Comparison between the visual analog scale (VAS) and the category ratio scale (CR-10) for the evaluation of leg exertion Int J Sports Med 1992 13 133 136 1555902 10.1055/s-2007-1021244

[B26] Kuorinka I Jonsson B Kilbom Å Vinterberg H Biering-Sørensen F Anderson G Jørgensen K Standardised Nordic questionnaires for the analysis of musculoskeletal symptoms Appl Ergon 1987 18 233 237 15676628 10.1016/0003-6870(87)90010-x

[B27] Dickinson CE Campion K Foster AF Newman SJ O’Rourke AM Thomas PG Questionnaire development: an examination of the Nordic musculoskeletal questionnaire Appl Ergon 1992 23 197 201 15676868 10.1016/0003-6870(92)90225-k

[B28] Beighton PH Solomon L Soskolne CL Articular mobility in an African population Ann Rheum Dis 1973 32 413 418 4751776 10.1136/ard.32.5.413 PMC1006136

[B29] Kaergaard A Andersen JH Musculoskeletal disorders of the neck and shoulders in female sewing machine operators: prevalence, incidence, and prognosis Occup Environ Med 2000 57 528 534 10896959 10.1136/oem.57.8.528 PMC1740005

[B30] Hoppenfeld S Zeide MS Orthopedic dictionary 1994 USA: Philadelphia

[B31] Bron C Franssen J Wensing M Oostendorp RA Interrater reliability of palpation of myofascial trigger points in three shoulder muscles J Man Manip Ther 2007 15 203 215 19066669 10.1179/106698107790819477 PMC2565638

[B32] Litchman HM Paslay PR Determination of finger-motion impairment by linear measurement JBJS 1974 56-A 85 91 4812170

[B33] Hoppenfeld S Physical examination of the spine & extremities 1976 New Jersey, USA: Prentice Hall

[B34] Constant CR Murley AH A clinical method of functional assessment of the shoulder Clin Orthop Relat Res 1987 214 160 164 3791738

[B35] Spitzer J Zaslaw N Orchestra 2011 Grove Music Online Oxford Music Online, Accessed Dec 25th, 2011 http://www.oxfordmusiconline.com (Direct article access for subscribers: http://www.Oxfordmusiconline.com/subscriber/article/grove/music/20402

[B36] Faulkner RR Career concerns and Mobility motivations of orchestra musicians Sociol Quart 1973 14 334 349

[B37] Johansson YL Theorell T Satisfaction with work task quality correlates with employee health Med Probl Perform Art 2003 18 141 149

[B38] Lusted MI Carrasco CL Mandryk JA Healey S Self reported symptoms in the neck and upper limbs in nurses Appl Ergon 1996 27 381 387 15677078 10.1016/s0003-6870(96)00030-0

[B39] Löfqvist L Pinzke S Stål M Lundqvist P Riding instructors, their musculoskeletal health and working conditions J Agric Saf Health 2009 15 241 254 19728547 10.13031/2013.27408

[B40] Schibye B Skov T Ekner D Christiansen JU Sjøgaard G Musculoskeletal symptoms among sewing machine operators Scand J Work Environ Health 1995 21 427 434 8824748 10.5271/sjweh.58

[B41] Ohlsson K Attewell RG Johnsson B Ahlm A Skerfving S An assessment of neck and upper extremity disorders by questionnaire and clinical examination Ergonomics 1994 37 891 897 8206057 10.1080/00140139408963698

